# Sector switching among histopathologists in KwaZulu-Natal, South Africa: a qualitative study

**DOI:** 10.1186/1478-4491-11-23

**Published:** 2013-05-30

**Authors:** Shaun D Ruggunan, Suveera Singh

**Affiliations:** 1 Discipline of Human Resources Management, University of KwaZulu-Natal, University Road, Durban, 4000, South Africa; 2 Programme for Industrial, Organisational and Labour Studies, University of KwaZulu-Natal, Mazisi Kunene Road, Durban, 4041, South Africa

**Keywords:** Histopathologists, South Africa, Push-pull, Health professional, Private sector mobility

## Abstract

**Background:**

The mobility of health care professionals from the public to private sector is prevalent in South Africa. However, literature on sector switching of clinical doctors remains limited. It is against this background that this study aims to make the labour market visible for histopathologists and identify the reasons for sector switching.

**Methods:**

This study is exploratory and descriptive. It uses qualitative methods, such as in-depth interviews, with 70% (n = 16/23) of the population of histopathologists in KwaZulu-Natal, South Africa. Lee’s (1966) push-pull theory is adapted to explain the pull sector switching behaviours of histopathologists. Interviews were recorded and independently transcribed. The narratives of the participants were coded to reflect the main themes that contributed to their sector switching behaviours.

**Results:**

Five key themes emerged as reasons for the mobility of histopathologists from the public to private sector in KwaZulu-Natal. The findings indicate that remuneration, working conditions, work flexibility, career pathing and autonomy of labour processes are the key drivers of this mobility.

**Conclusions:**

Histopathologists provide a core function in the health care chain. However, their invisibility in academic discourse in both public health and human resources for health indicates the paucity of research undertaken on the importance of these specialists in the health care chain. This is especially significant in developing countries like South Africa, where there is a dearth of these specialists. This study, while exploratory, aims to open a dialogue to better understand their reasons for sector switching and, hopefully, inform policies on training, recruitment and retention of these specialists.

## Introduction

This article aims to elucidate the factors that contribute to histopathologists’ mobility from the public to private sector in KwaZulu-Natal, South Africa. The shortage of histopathologists is a growing problem, both globally and nationally [1-7]. Empirical work on labour market mobility in the health care sector is dominated by studies of clinical health practitioners, while there is a dearth of empirical work on local labour market mobility of South African medical laboratory specialists. This article addresses the empirical gap in South African and international literature by rendering visible the labour market for histopathologists in KwaZulu-Natal. This visibility shows that histopathologists are an essential link in the clinical health care chain. As such, their retention in the public sector needs to be prioritized.

This study focuses on possible pull factors, such as salary rates and working conditions, as reasons for the sector switching of histopathologists, instead of push factors, such as natural disasters, which do not apply to this case study. Based on Lee’s (1966) work and subsequent updates, five pull factors emerged as reasons for the mobility of histopathologists out of the public sector and into the private sector. These are: 1) more attractive remuneration; 2) better working conditions and environments; 3) a higher level of work flexibility; 4) improved career mobility; and 5) more autonomy of labour processes.

Theoretically and empirically, sector switching is limited in the discourse on public sector organizations [8]. In order to better understand the local mobilities of medical specialists, including histopathologists, a more contextual and qualitative account of labour markets is needed.

South Africa is one of the countries that have what is termed a ‘dual or fragmented health system’ [9,10]. The South African health care system comprises the public sector and private sector, with the former encompassing government health institutions to primarily serve the majority of the population, while the private sector comprises for-profit organizations and individuals for insured patients and those who can afford health care on an ‘out-of-pocket’ basis [9].

The public health workforce is diverse and its key responsibility is to provide core public health activities, regardless of their organizational base [11]. Strikingly, the public sector accounts for only 20% of total health expenditure in South Africa, despite catering for 82% of the population [9]. This is in contrast to the private sector which serves 20% of the population, but consumes up to 60% of the country’s health expenditure [9,10,12,13]. The empirical work indicates that South Africa has inherited a disjointed health care system reflecting disparities in health care spending regarding the distribution of health professionals, access and quality of care between and within provinces, races, urban and rural areas, and between public and private health care sectors.

Empirical work has demonstrated that the public health care sector is under-resourced and over-used [9-12,14-17]. The South African public health care sector appears inefficient and ineffective with regard to providing accessible, affordable and suitable health care [15,18,19]. The increasingly large private sector, by contrast, has a good reputation and is recognized for its world-class facilities [9,10,18,20].

While there are instances where the public and private sectors do interact and cooperate by sharing resources and contracting out to each other, these arrangements are ‘loose and unstructured’ [21]. Despite the improvement in health care spending among the disadvantaged provinces in South Africa, the average real per capita health care expenditure by the state has only increased at an annual rate of 0.3% since 1998 [22].

This is compounded by the fact that the majority of South African medical practitioners practise in the private sector [10,23]. They comprise 67% of general medical practitioners and 75% of medical specialists [10]. The ratio of public to private health workers has deteriorated from a ratio of 12.0 public sector health workers per 10,000 population to 10.7 per 10,000 population [10]. During the early 1980s, approximately 40% of doctors worked in the private sector. A decade later, 62% of general practitioners and 66% of specialists worked in the private sector [12]. South African health care workers are leaving the public sector and joining the private sector where the working environment is more ‘comfortable’ and ‘affluent’ [24]. This is an indication of the unequal distribution between the public and private sectors.

In South Africa, there are officially 245 histopathologists, 115 haematologists, 28 virologists and 115 chemical pathologists [19]. However, based on the fieldwork for this study, these official statistics have proved inaccurate. The register may be unintentionally inflating the labour market statistics. For example, the register lists medical laboratory doctors who are actually working full-time outside South Africa but who have also maintained their registration on the Health Professions Council of South Africa (HPCSA) register. The register also still reflects those who have retired from practice but not withdrawn their registration. Without being able to disaggregate the labour market statistics in this way, it is difficult to agree on the exact number of laboratory doctors who are practising full-time in South Africa.

The labour market for histopathologists, both nationally and provincially in KwaZulu-Natal, is racially skewed. Nationally, the discipline is white South African and male-dominated, and in KwaZulu-Natal it is Indian South African and male-dominated. This reflects historical apartheid legacies of training and employment. For example, the national labour market for histopathologists is comprised of 5% black South African, 9% Indian South African, 61% white South African, with 25% of pathologists remaining racially unclassified. The most likely reason for this last cohort being racially unclassified is that they represent the 25% of ‘missing’ pathologists from the country who are, in fact, practising overseas and hence difficult to trace and classify racially. In KwaZulu-Natal, the specialty is male-dominated and only 7 of the 23 KwaZulu-Natal pathologists are female. This trend is also evident at a national level, with only 90 of the 245 (37%) histopathologists being female.

Extrapolating from national South African statistics, which locate 67% of all medical specialists in the private sector, one can surmise that there is a similar trend for medical laboratory specialists [25].

## Methods

### Adapting Lee’s push-pull theory to explain sector switching

While Lee developed the push-pull theory to explain international migration, it provides a useful framework to understand local mobilities of medical doctors between public and private sectors. Most of the literature on this mobility has applied the push-pull theory to explain global labour migration behaviours, but in the South African literature, its application to sector switching is limited. This study attempts to adapt the theory by demonstrating its application in understanding sector switching of histopathologists in KwaZulu-Natal.

### Study design

Since this is the first empirical research into the labour market for histopathologists in KwaZulu-Natal and South Africa, an exploratory and descriptive case study approach was used. In-depth interviews were the main data collection tool. The interview instrument was developed after a pilot interview with two histopathologists. The process of conducting the interviews developed iteratively.

### Population and sample

The population for this study was 23 histopathologists employed in the public and private sectors in the province of KwaZulu-Natal. Purposeful sampling was used. The key criterion was that all participants had to be qualified histopathologists employed in the public or private sector in KwaZulu-Natal. Of the population of 23, a sample of 16 was realized.

### Data collection

Interviews were conducted in English by a team of four experienced researchers with training in the interview process. The 16 interviews were conducted off-site in a University seminar room. Interviews averaged 1 hour in length and followed a pre-prepared, open-ended interview schedule. All interviews were conducted in 2011. The schedule served as a guide only, to allow for probing questions which were usually asked to seek clarification from respondents about their responses. Interviews were recorded digitally, with participants’ permission. Audio interviews were thereafter transcribed by a professional transcribing service and reviewed by the research team to ensure they correctly reflected the content of the interviews.

### Data analysis

Qualitative thematic content analysis was conducted which identified prominent themes from the interviewees’ narratives. Through the process of listening to and reading the interviews, we were able to generate a sense of how and why interviewees decided to switch sectors. Thematic analysis was then employed, whereby quotes from the interview data were coded into themes. The transcripts were constantly compared with each other to see what patterns or themes emerged across the interview data. The coding of data into themes was undertaken independently by each team member. Team members would then meet collectively and compare their codes. When there was no consensus on codes, codes were either expanded or refined. Given that the data was voluminous, matrices and tables were used to organize quotes from the interviews and generate codes. Matrices also allowed for a condensation of the interview data. This helped the researchers decide how the themes were to be coded. The summarized data also facilitated the comparison of the research teams’ independent coding of the data. Through this process the reasons for the mobility between sectors were identified.

### Ethical clearance

Ethical clearance was obtained from the University of KwaZulu-Natal’s Ethics Committee. The ethical clearance number is: HSS/1187/010. All participants signed an informed consent form and were informed that they could withdraw from the study at any point.

### Findings: participants’ perspectives on why sector switching occurs

In this study, a purposeful sample of seven histopathologists from the private sector and nine histopathologists from the public sector was selected. They represent a total of 16 of the 23 histopathologists employed in KwaZulu-Natal (Table 1).

**Table 1:** Participants’ demographics

**Gender, number**	**Race, number**	**Sector, number**
Female, 2	White South African, 1	Private, 7
Male, 14	Black South African, 2	Public, 9
	Indian South African, 13	

#### Remuneration: histopathologists are paid better in the private sector

One of the main themes that emerged from the participants’ accounts on why sector switching occurs is that of remuneration. There was a strong sense among participants that salaries and other forms of remuneration for histopathologists are much higher in the private sector than in the public sector. The majority of participants concurred that, while salary rates may not be the only catalyst for sector switching, it remains a powerful motivator. The two following quotes exemplify the narratives expressed by participants in this regard:

And I think one of the biggest [challenges] is the remuneration because the public sector cannot compete with the private sector. (Interview: participant 1)

Pay is lower and they [are] struggling to retain people in the public sector. (Interview: participant 3)

This sentiment is echoed by a third participant:

… it is common sense to move out of the public sector, as the private sector pays higher salaries than the public sector. There are attempts by the public sector to improve salaries but at this point it’s far, far more lucrative to be employed by the private sector. (Interview: participant 5)

However, as much as higher salaries have motivated histopathologists to move to the private sector, the interview data revealed that the difference between remuneration in the sectors is not that big. Participants 1, 2, 3, 4 and 5 indicated that annual salaries were 20% higher in the private sector. Interviewees also reported that in certain instances, by working overtime, public sector histopathologists can theoretically enjoy higher salaries than their private sector counterparts.

When participant 7 was asked why he recently shifted to the private sector, he indicated the following:

… well you know in a country like South Africa where we have to pay a fortune for private schooling and private health care, higher salaries also help me in taking care of my family and children. If you are a family man then it’s not possible to raise a family on a public sector salary. (Interview: participant 7)

Two histopathologists in the private sector expressed a desire to do more academic and research-related work, which is not always possible in the private sector. However, the lure of better salaries in the private sector means that there is no flow back from the private to the public sector, despite public sector work being more intellectually stimulating, according to interviewees. As one participant stated:

We always get people saying that they miss the academic environment but what people say and what they actually do doesn’t result in the CV coming back to our desk. No, I miss the academy, the journal clubs, the seminar presentations, but at that stage, those people are close to retirement or partners and the state cannot afford even an equivalent salary package and it’s not worth their while to come back. (Interview: participant 6)

Highly skilled medical professionals expect a certain rate of financial reward, either through gross salaries or incentives. The findings on remuneration indicate that even though the salary discrepancy is not great, it is significant enough to act as a pull factor from one sector into another.

#### The working environment: histopathologists enjoy better working conditions in the private sector

A second strong narrative to emerge from the interviews was that of the differential in working environments in the public and private sectors in KwaZulu-Natal. Participants indicated that working conditions and the working environment have a major impact on mobility between sectors in several ways. This is in keeping with work by Hansen (2011) and Brugha et al. (2010), which revealed that the public to private migration of health workers in Zambia [26]was caused by increased workloads and adverse working environments.

Participants’ responses demonstrate that the public sector working environment differs from the private sector and is a reason for mobility. For example, the level of communication or interaction between the patient, physician, primary referring doctor and pathologist is higher in the private sector for histopathologists than in the public sector. If working time flexibility is restricted, pathologists are more likely to seek a new working context, which often involves sector switching. In the private sector, there is the option to work part-time, unlike in the public sector (participants 3, 4, 5, 6 and 7). In the public sector, staff are required to work a full shift, with no option of undertaking private sector work for extra remuneration (participants 10, 11, 12, 13 and 14). Positions in the private sector are said to come with laptops, travel allowances and cell phone allowances, which are added attractions (participants 3, 5 and 7).

As one of the participants indicated:

… because I mean the working environment is so much different, forget the financial rewards, that is obviously a big factor, but in the public sector one of the biggest problems there is that you have a very poor communication between the patient, the physician, the primary referring doctor and eventually the pathologist. As a pathologist you are like a super specialty, you are the last port of call kind of thing. So when you get a specimen coming to you, you are almost looking at it in isolation. There is very limited interaction. Whereas here you are on the phone, or even the doctor or the patient visits you, like you are doing now, and you get a well-informed background to the patient before you look at the specimen. There you work in kind of isolation. (Interview: participant 6)

Participant 13 stated that if the environment was not conducive, then one would have to leave. Long working hours in the public sector were also identified as a reason for moving to the private sector. This participant further noted that:

Firstly, it’s the working conditions in terms of the working hours. In the public sector, because of the volume of work that we have, our working hours are quite long. So we actually extend beyond what is expected of it because we just need to complete the work. (Interview: participant 13)

Participant 16 explained:

I worked as a sessional doctor for approximately 3 years after I had a twin pregnancy. Then when I was ready to go back into full-time employment, I had another child. A limited working day became critical. The public sector was not able to accommodate me at that point. (Interview: participant 16,)

Further, the study found that the working environment in the public sector is largely ‘supervisor-dependent’ (participant 9). A supervisor-dependent management style is a pull factor, according to participants interviewed. As one participant indicated:

So it’s a huge thing, so I think largely it’s supervisor-dependent. If the supervisor wants to micromanage and things like that, you are not going to get happy staff. So I think therefore people move to private as well because they are not micromanaged. (Interview: participant 9)

An encouraging supervisor, according to a participant would:

… create a more conducive and positive working environment that increases productivity and cooperation. (Interview: participant 10,)

This can influence the decision to move sectors. One participant explained that, had there been a different head of department, he would not have left the public sector. He said that he wanted to grow in terms of his career without any ‘obstruction’. Management styles also differ between the public and private sectors (participant 12). He elaborated:

But when you come into private practice it’s a different ball game. You have got to manage. You manage perceptions, you manage attitudes and you manage people … if you treat someone badly they will have to leave … you just listen and be compassionate … if you manage people’s emotions at work, you will go far as a leader. And in the state sector, you are a number, you are not a person. You are just a number. (Interview: participant 12)

#### Autonomy: histopathologists have more freedom in the private sector

A third narrative to emerge from the data is that of professional autonomy or freedom. For participants, autonomy in labour processes is a powerful catalyst for mobility out of the public sector. Participants used words such as ‘independence’, ‘freedom’, ‘choice’ and ‘self-management’ to demonstrate the importance of autonomy to them as professionals. Job autonomy refers to the extent to which a job provides freedom, independence and, most importantly, discretion in work content and methods of working, as well as the pace at which one chooses to work. The public sector is said to have a high degree of rules and regulations with a steeply hierarchal structure, as well as numerous protocols and red tape, which lead to frustration among professionals (participants 1, 2, 3, 4, 5, 6, 7 and 8). This sector is also perceived as having a more rigorously controlled working environment compared with that of the private sector (participants 10, 11 and 12). Both autonomy and flexibility are needed for career advancement, and these are found to a greater degree in private practice. Highly skilled professionals, such as histopathologists, expect a level of recognition and responsibility on reaching a certain point in their careers and, as argued by the above participants, they should be allowed to act at their own discretion. The quote below captures the frustrations of histopathologists working in the public sector:

… you know, I did not train for almost a decade to be micromanaged and treated like a child. Even people in factories have more freedom than we do in the public sector. My colleagues in other specialties and even my friends in completely different jobs cannot believe the level of micromanagement and control. Part of it is institutional and part of it is managerial style. How am I supposed to grow as a professional? (Interview: participant 15)

In addition to the lack of autonomy is the excessive workload faced by pathologists in the public sector. Participant 14 stated:

I know people who were very unhappy at work. And we have got some pathologists who resigned from there [public sector], senior people, and came to private practice and it’s because of the work environment and especially the high workloads. We have to do up to three times the workload of colleagues in private. (Interview: participant 14,)

Participant 16 cautioned, however, that we should not extrapolate her comments to mean that the public sector is managed in the same way nationally. She stressed that, nationally, departments differ in managerial styles:

Maybe in Cape Town their experience is different, but for me, here in Durban, public [sector] is hell … It could have been different. (Interview: participant 16)

#### Flexibility: histopathologists enjoy more working time flexibility in the private sector

A fourth theme to emerge from participants’ accounts and related to the previous theme of autonomy is that of flexibility of working time. The findings indicate that it is the relative degree of flexibility that influences their decisions to remain in the public sector or not. The quote below captures the views of participants from the public sector:

Well as it is we are fixed to our desks. It’s impossible to have children and do school pick-ups and drop-offs … because we have to clock in and clock out. It’s just very demoralizing that as a professional I can’t manage myself. My family does not understand how a doctor cannot be trusted to do her work. Yeah I understand the deadlines and such but having to explain every minute of your time out of the office, even for lunch is undermining. (Interview: participant 11)

The degree of flexibility of working hours differs greatly between the two sectors (participants 11, 12 and 13). Working hours for medical laboratory specialists in the private sector are more flexible than in the public sector, as indicated by participant 6:

Well here in the private laboratory, I am treated like a skilled professional; there is a high level of trust. I get my set cases for the day and I am responsible enough to complete those cases, even if I have to run domestic errands or come in later in the morning. At first I was confused when I left the public sector and suddenly no one was monitoring my comings and goings. I realized that as long as the work gets done, my boss is happy. The irony is that we are more profit-driven in the private sector but management allows us full flexibility to control how we work during the day. (Interview: participant 6)

Participant 4 explained that, in the private sector, one might leave once one’s work is done, whereas even if that option exists in the public sector, one may not be able to exercise it due to the onerous workload. Participant 10 from the public sector indicated that flexibility was possible under certain circumstances:

Well I think if you are closer to retirement age, then the public sector is more open to negotiating flexible working hours with you … (Interview: participant 10)

This view was echoed by participant 12 who has worked both in the public and private sectors. He stated that, while flexibility is possible in the public sector under certain circumstances, these types of arrangements are:

… much more easily negotiated in the private sector. (Interview: participant 12)

Participant 13 from the public sector indicated that:

The reason they have also gone into private is they were offered what they wanted and most of them are doing half day jobs as opposed to full day jobs. (Interview: participant 13)

Further findings from the interviews demonstrate that, in the private sector, there are fewer rules about reporting for and leaving work at set times (participant 1) or what participant 1 refers to as ‘clocking in’. The level of flexibility in the private sector is said to make the working environment more ‘conducive’ to working (participants 1, 4 and 6). In the private sector, one is even able to work from home (participant 7). Whereas a private sector pathologist can sign off work in the comfort of their own home, this option is non-existent in the public sector, despite the technology being available to do so (participants 1, 2, 3 and 7).

Many histopathologists, especially those with families, have opted to move into the private sector because the working hours are more flexible (participant 4). Participant 6 explained that he needed more family time, which was not possible in the public sector where he worked until nine or ten o’clock at night. Participant 3 described the working hours in the public sector as ‘ridiculous’ (participant3) Furthermore, the private sector offered the participant a half day post with a competitive salary, which enabled more family time. One participant requested a half day job in the public sector; however, the cut in her salary was dramatic, causing her to move to the private sector (participant 16). In fact, her salary would have been halved, whereas when she went into the private sector she was offered one and a half times the full-time salary she had earned in the public sector (participant 16).

Both male and female interviewees (participants 1, 2, 3, 12, 13, 14 and 16) contended that flexible work time is equally important for men and women. Flexible working hours are available to both genders in the private sector and are a compelling reason for pathologists to transfer into private practice.

#### Career pathing: histopathologists have better career pathing in the private sector

A common thread through the various narratives of the participants was their implicit and explicit references to career pathing and career mobility. Their narratives demonstrate respondents’ beliefs that the public sector offers minimal career pathing opportunities compared with employment in the private sector.

That’s an interesting question … my experience has been that unless I wanted to become head of department there was no other career path available to me. Now that I am in private employment, I can decide to have a career track towards partnership, or focus more on marketing of the laboratory, or pursue research-based projects on my own. I’m still young so I haven’t decided how I want to develop but there are, for sure, many, many options available to me here [in private sector] than at my previous employer. (Interview: participant 4)

Long-term career prospects are better in the private sector than in the public sector. As one participant from the public sector expressed:

They have frozen posts … the people who qualified are still stagnant, not moving up, no job offers. So, if jobs are frozen, people are going to leave. They are not going to stay at that level, registrar or whatever level, because they are now qualified. There is a need for more consultants but they are not opening it up. So there is an exodus at the moment. People are leaving and it is a lot. (Interview: participant 15, 2011)

Restrictions on upward mobility in the public sector may cause professionals to leave. The private sector is said to offer a sense of business-oriented growth as well as ‘professional stimulation’, which may be seen as a reason to seek employment in this sector (participant 13). The findings are summarized in Table 2.

**Table 2:** Pull factors from the public to the private sector (n = 16)

**Reasons to migrate out of the state sector**	**Number of interviewees who cited this reason**	**Percentage of interviewees who cited this reason (%)**
Remuneration	16	100
Working conditions	14	88
Autonomy of work	14	88
Flexibility of working time	13	81
Career pathing	10	63

## Discussion

Findings from the narratives of the interview data reveal five themes, which are congruent with Lee’s concept of pull factors. The analysis for this study has restricted itself to understanding the pull factors that drive histopathologists from the public sector into the private sector. The reasons for the sector switching of histopathologists have to be contextualized within the broader context of the global crisis in human resources for health. According to Lee, push factors drive people to migrate, whereas pull factors attract them to new work locations [27-31]. Push factors include, but are not limited to, a lack of decent employment opportunities, lower salaries, poor working conditions, poor infrastructure and technology, a lower social status, repressive governments, pollution, natural disasters, and discrimination. The push-pull theory has been successfully used to explain the migration of skilled workers from sending to receiving countries. Collectively, work by these researchers [27-31] demonstrates that Lee’s theory remains a relevant and vibrant way of theorizing labour migration of skilled workers. The findings demonstrate that, while Lee’s theory was conceptualized to explain global labour migrations, it can also be adapted to explain the mobility of histopathologists between the public and private sectors in KwaZulu-Natal, South Africa.

The findings of this case study are in keeping with larger scale studies by the International Organization for Migration (IOM) [32], which identified similar reasons for the mobility of health personnel from the public sector to the private sector. These reasons include better salaries, enhanced working conditions, greater career advancement opportunities, and streamlined and responsive institutional procedures and regulations [32]. The findings are also congruent with the studies of Beckering and Brunner [3], Guidi and Lippi [33], and Plebani [34], which demonstrate that key reasons for the shortage in the public sector of medical laboratory specialists, including histopathologists, are early retirement, salary dissatisfaction and job dissatisfaction. For instance, Plebani [34] observes that medical students are reluctant to pursue careers as laboratory specialists due to public sector and state inadequacies in recruiting registrars, the escalating costs of training in the public sector, diminishing budgets for public sector hospital laboratories, poor wages, and a lack of career growth and advancement in the public sector.

Most participants identified remuneration as a core concern. Its remains unlikely that public sector remuneration for histopathologists will be increased; the parastatal body that employs histopathologists in South Africa, the National Health Laboratory Service (NHLS), was haemorrhaging money in 2012 [35] to the extent that a national crisis was declared for laboratory services in South Africa [35]. An implication of the financial crisis experienced by the NHLS is that it potentially does not have the funds to increase salaries for histopathologists in the public sector. According to two participants (participants 11 and 13) there have been instances, when histopathologists have resigned, where the state has intervened to match private sector salaries. However, the process of making the decision to match salaries is onerous. This decision requires a lengthy process, involving an executive committee as well as the Chief Executive Officer of the NHLS. Due to the less complex organizational structure of laboratories in the private sector, it is a much easier process for a private laboratory to match a medical specialist’s salary.

This finding is in keeping with evidence that suggests that private sector health care workers, such as doctors, nurses and allied health professionals, are offered more attractive remuneration packages [10,20,36]. Moreover, Pillay’s study [9] revealed that remuneration and poor working conditions have been causes of dissatisfaction for nurses in the public sector. Remuneration specifically, has been a reason for the movement of nurses to the private sector. Private sector nurses were generally satisfied, felt a sense of belonging in the communities where they worked and felt that their working environment was safe. By contrast, public sector nurses were generally dissatisfied [9]. Pillay’s findings contradict general management literature, which indicates that public sector satisfaction has improved over the years relative to the private sector [9].

Coovadia et al. [12] made mention of several ‘unfortunate’ policy decisions, such as the voluntary severance packages offered to public sector personnel, which resulted in the movement of skilled staff out of the public sector and into the private sector, international agencies or even retirement. In the South African context, salary increases could stem the flow from the public to the private sector [37]. A study of the whereabouts of graduates of the University of the Witwatersrand, Johannesburg, showed that 64% of those working in the private sector said that ‘income-generating potential’ was their reason for working in this sector [37]. This is in keeping with Lee’s original premise that poor remuneration is a push factor.

While attractive salaries are a key pull factor, the literature demonstrates that it is not the only factor. This supports the findings of this study which showed that public to private sector mobilities are the outcome of a myriad of pull and push factors. For example, the National Human Resources (NHR) plan (cited in Breier and Erasmus [23]) states that salary is not the only factor influencing the migration of South African doctors. Working conditions and excessive workloads are also identified (Department of Health, cited in Breier and Erasmus [23]). The study by Bezuidenhout et al. in 2009 [38] on the emigration of South African qualified physicians makes reference to the push-pull theory, which reinforces its relevance. This study showed that salaries were not the sole factor that caused the migration of physicians. Approximately 86.2% of respondents left South Africa for financial reasons and 79.3% for improved job opportunities, while 58.6 percent wanted to experience something new, based on personal preferences, and 51.7% left due to high levels of HIV/AIDS [38]. The main findings from this study support Hansen’s [8] assertion, that higher remuneration in the private sector remains a pull factor for those in the public health system.

The next theme that emerged from the findings was the issue of working environments which operate as both push and pull factors. Narasimhan et al. [39] and Ashmore [36] maintain that the quality of the public health work environment is deteriorating along with education and training, which lack appropriate funding. In addition, dissatisfaction, limited professional development and recognition, as well as strained relationships with co-workers and peers were also cited, although none of the participants in this study cited relationships with peers as either a push or pull factor. Instead, respondents placed greater emphasis on their relationships with their line managers or heads of department. Moreover, Pillay’s study in 2009 [9] revealed that poor working conditions have been a cause of dissatisfaction for nurses in the public sector. Private sector nurses, by contrast, were generally satisfied, felt a sense of belonging in the communities where they worked and felt that their working environment was safe. By contrast, public sector nurses were generally dissatisfied [9].

Another issue that might motivate medical laboratory specialists to leave the public sector is the way they are managed. Participants revealed that people who work in the public sector are not treated as professionals, despite having achieved a particular status. The human resources and industrial psychology literature demonstrates that a healthy and motivated workforce is vital in the retention of health workers in the public sector [39]. There is clearly a deficit in effective managerial and leadership styles in the public sector for histopathologists in KwaZulu-Natal. The autocratic managerial style in the public sector stifles autonomy, flexibility and career pathing of histopathologists. In so doing, productivity levels, trust levels and job satisfaction levels decline rapidly. Ashmore’s study in 2013 [36] on working conditions in South Africa’s public and private health care sectors is congruent with the findings of this study, and that of Pillay’s 2009 study [9].

Empirical work demonstrates that high levels of job autonomy can lead to increased productivity and flexibility, and enhance an organization’s ability to retain workers [40,41]. Histopathologists need a high degree of flexibility when they reach a certain stage in their career. Involvement in management and decision-making as well as supervisor support for job success are an integral part of achieving efficiency in any work environment [40]. Thus, it is essential in any working environment to display a level of respect and acknowledgement of staff members in order to maintain a healthy and balanced workforce. Studies by Kline [42], Narasimhan et al. [39], Pillay [9] and Ashmore [36] found the key factors leading to dissatisfaction to be ‘non-supportive work environments’, as well as increased workloads.

Chen et al. [43] highlight the importance of countries across the globe improving their working environments by way of reinforcing good practices to enhance the management of resources, management of professionals, provision of sufficient access to supplies and facilities, and development of incentives, both financial and non-financial, to retain and encourage health workers. Narasimhan et al. [39] point out that unsupportive working environments and low compensation ‘demoralize’ workers and may result in a shift to private practice, either in the short-term or on a permanent basis.

Working time flexibility is a non-financial incentive that acts as a pull factor for histopathologists into the private sector. The literature on flexibility notes that it is associated with increased job satisfaction and greater work commitment [40,41]. Flexibility arrangements that are family-supportive would increase job satisfaction [40,41]. For example, workplace flexibility demonstrably increases retention and influences employees to remain with their current employer [40,41]. Ashmore’s study in 2013 [36] on job satisfaction of doctors in the public and private sector in urban South African hospitals confirms the empirical studies in the literature, that non-monetary benefits, such as flexibility, are important to medical doctors.

Participants’ accounts also revealed a concern with career pathing and the lack of career pathing opportunities in the public sector. Allsop et al. [31] emphasize that the decision to migrate from one employer to another is often associated with reasons such as the desire to gain additional experience. For example, Maistry (cited in Hudson [44]) reports that for a cytologist returning to South Africa after having worked 3 years in Saudi Arabia ‘meant taking a few steps back professionally’. Hudson [44] adds that one of the reasons that newly qualified specialists move into the private sector or leave the country is the unavailability of consultant posts in the public sector. It is evident from the interview data that histopathologists need to have a sense of a future career path. That path could develop into several routes, including a managerial route, research route or a more operational route. Histopathologists want career choices or tracks to be available to them. Participants expressed a desire to grow professionally beyond their diagnostic skills and roles. It is frustrating for them that these career pathways are not available in the public sector. This is compounded by their having no control over their careers in the public sector. If histopathologists are to be retained in the public sector, then clear career pathing opportunities need to be made available for them. While in Ashmore’s study in 2013 [36] medical doctors cited team work, more academically rewarding work, and a sense of service, relevance and contribution to the greater public good, as reasons why they remain in the public sector, no such sentiments were echoed in the accounts offered by the histopathologists interviewed for this study.

The mobility of medical laboratory specialists between sectors has a number of consequences, not just for themselves, but for those who are left behind. Firstly, as previous research shows, when some workers migrate, the remaining workers have to cope with the workload [3]. In the case of histopathologists, challenges are experienced at all levels of the laboratory. This means that the remaining pathologists are subjected to an escalation in stress and strain [3]. Laboratory staff are also affected, including clerical staff and technologists who work for the pathologists. Therefore, there is an increase in workload and stress for them as well. This is confirmed by studies that highlight that the shortage of medical laboratory specialists results in the existing workforce having to do the same volume of work that a fully staffed laboratory would have to do [3]. They are required to maintain the same turnaround times, leading to exhaustion, burnout and the increased likelihood of errors [3,4,45]. This assertion is borne out by the findings of this paper that histopathologists in the public sector are experiencing an increase in the volume and intensity of their workloads.

Secondly, if unfettered sector switching continues, then there is a danger, as has already occurred in some parts of Africa, that the entire public health system may collapse [24].

Thirdly, a stressed public sector laboratory service is inefficient, having profound consequences for patient health care. This is a challenge for many laboratories in Sub-Saharan Africa. A profound consequence of the lack of capacity is that misdiagnosis commonly occurs [46].

Finally, given the disconnect between the public and private sectors in South Africa, collaboration between sectors is viewed with suspicion and is the exception rather than the norm. A substantive way of dealing with the crisis of sector switching may be to formalize private-public sector cooperation.

## Conclusion

The main strength of this study is that it begins to address the empirical gap in the literature on the human resources crisis facing laboratory medicine in Sub-Saharan Africa. The study secured a high participation rate of 16 of the 23 histopathologists employed in KwaZulu-Natal. Further, the profession requires advocates on the continent to render visible the importance of these specialists to the chain of health care. However, given the focus on KwaZulu-Natal only and the nature of qualitative research, the findings cannot be generalized to other provinces in South Africa. It is hoped that this exploratory study into sector switching and the human resources crisis facing histopathologists in KwaZulu-Natal opens up the field for further research into this much neglected medical specialization. Such research may lead to important human resources policy interventions for the training, recruitment and retention of histopathologists.

## Abbreviations

CV: Curriculum vitae; HPCSA: Health professions council of South Africa; IOM: International organization for migration; NHLS: National health laboratory service; NHR: National human resources.

## Competing interests

The authors declare that they have no competing interests.

## Authors’ contributions

SR conceived and designed the study. SR analysed and interpreted the data. SR and SS drafted the manuscript. Both authors read and approved the final version of the manuscript.
